# Frequency Locking Method for Frequency Standards Based on Diamond NV Centers

**DOI:** 10.3390/s26061777

**Published:** 2026-03-11

**Authors:** Shiyu Guan, Bingfeng Sun, Qiyuan Jiang, Yuxiao Wang, Xubo Liao, Jie Yuan, Yi Zhang, Zhongqi Tan

**Affiliations:** 1 College of Advanced Interdisciplinary Studies, National University of Defense Technology, Changsha 410073, China; guanshiyu0211@outlook.com (S.G.); sbf3375038@163.com (B.S.); qiyuanjiang1990@163.com (Q.J.); waltonbryant@163.com (Y.W.); xuboliao@outlook.com (X.L.); jieyuan@nudt.edu.cn (J.Y.); 2 Nanhu Laser Laboratory, National University of Defense Technology, Changsha 410073, China; 3 The No. 32201st Troop of PLA, Baicheng 137000, China

**Keywords:** diamond NV center, quantum frequency standard, ODMR, frequency locking, Allan deviation

## Abstract

In this study, frequency locking technology is investigated for high-stability microwave frequency standards based on diamond nitrogen-vacancy (NV) centers. Conventional locking methods typically utilize the side peaks induced via Zeeman splitting; however, this approach renders the frequency output highly susceptible to ambient magnetic field fluctuations. To address this limitation, a robust frequency locking method based on the central peak of the Optically Detected Magnetic Resonance (ODMR) spectrum is proposed. By systematically optimizing the bias magnetic field, the proposed method exploits the central peak’s inherent insensitivity to magnetic field variations and its narrower linewidth in environments with weak magnetic fields, thereby enhancing the quality factor of the frequency discrimination curve. The experimental results demonstrate that the proposed scheme achieves closed-loop locking of the 2.87 GHz microwave frequency, reaching short-term frequency stability (Allan deviation) of 1.73 × 10^−7^ at 200 s. Comparative tests under gradient magnetic fields further confirm that central-peak locking significantly suppresses frequency drift compared to side-peak methods. This study provides a vital technical pathway for the development of miniaturized, interference-resistant solid-state quantum frequency standards.

## 1. Introduction

High-precision, portable time–frequency standards are critically demanded in advanced navigation and communication systems [1,2,3]. While chip-scale atomic clocks (CSACs) based on Rubidium (Rb) [4,5,6] or Cesium (Cs) atomic vapor cells [7] are widely commercialized, their reliance on high-temperature heating increases power consumption and warm-up times, and buffer-gas collisions limit further long-term stability improvements. To address these bottlenecks, research has increasingly focused on solid-state quantum frequency standards; these devices are particularly promising due to their lack of warm-up requirements, resilience to environmental interference, and potential for seamless integration. As a solid-state point defect capable of maintaining long spin coherence times at room temperature, the nitrogen-vacancy (NV) center in diamond has recently emerged as a prominent topic in the fields of quantum sensing and precision measurement [8,9]. Thanks to its stable lattice structure and unique Optically Detected Magnetic Resonance (ODMR) readout mechanism, the NV center has been widely applied in various emerging fields, including high-sensitivity magnetometry [10,11,12,13], nanoscale thermometry [14,15], electrometry [16], and quantum computing [17]. In the domain of frequency standards, solid-state frequency source schemes based on NV centers are regarded as strong competitors for next-generation chip-scale atomic clocks (CSACs) [18], owing to advantages such as radiation hardness, the absence of buffer gases, and compatibility with semiconductor manufacturing processes. Hodges et al. first proposed the theoretical concept of utilizing NV-center ensembles as time–frequency standards [19]. The physical principle underlying this method relies on the NV center’s intrinsic zero-field splitting (ZFS) between its ground-state electron spin levels ($m_{s}=0$ and $m_{s}=\pm 1$). At room temperature, this ZFS provides a highly stable microwave transition frequency naturally pinned at approximately 2.87 GHz, making it an excellent atomic frequency reference. The concrete technical approach involves continuously probing this intrinsic transition via ODMR and extracting an error signal to lock a local microwave oscillator to the resonance dip, thereby stabilizing the final clock output.

However, engineering NV centers into high-stability frequency sources entails a core technical challenge: the electron spin resonance frequency is extremely sensitive to magnetic fields (gyromagnetic ratio of $\gamma_{e}\approx 28$ GHz/T). In conventional locking schemes that utilize the “side peak” induced by Zeeman splitting, geomagnetic fluctuations or environmental electromagnetic interference in the order of microteslas (μT) directly translate into significant frequency drifts, severely compromising the clock’s stability. Because the performance of an atomic clock fundamentally depends on the constancy of its macroscopic reference frequency over time, any environmental magnetic noise directly causes the locked frequency to fluctuate. This uncontrolled frequency drift mathematically manifests as a severe degradation in the long-term stability (Allan deviation), causing the clock signal to lose its precise timing capability. To address the aforementioned issue of magnetic sensitivity, this study proposes and validates a robust microwave frequency locking technique based on the ODMR “central peak.” By leveraging the fact that the central peak—formed by the overlapping of degenerate energy levels under weak magnetic fields—is insensitive to magnetic perturbations, we suppress the interference of external magnetic field fluctuations without the need for complex magnetic shielding. The experimental results indicate that, compared to the conventional side-peak locking scheme, central-peak locking improves the short-term frequency stability (Allan deviation) of the system by a factor of 4, significantly enhancing its robustness in non-laboratory environments. This study provides a critical technical foundation and experimental evidence for the development of low-cost, miniaturized, and portable solid-state quantum frequency sources capable of resisting magnetic interference.

## 2. Principle

### 2.1. Theoretical Model of the NV Center

**Figure 1:**
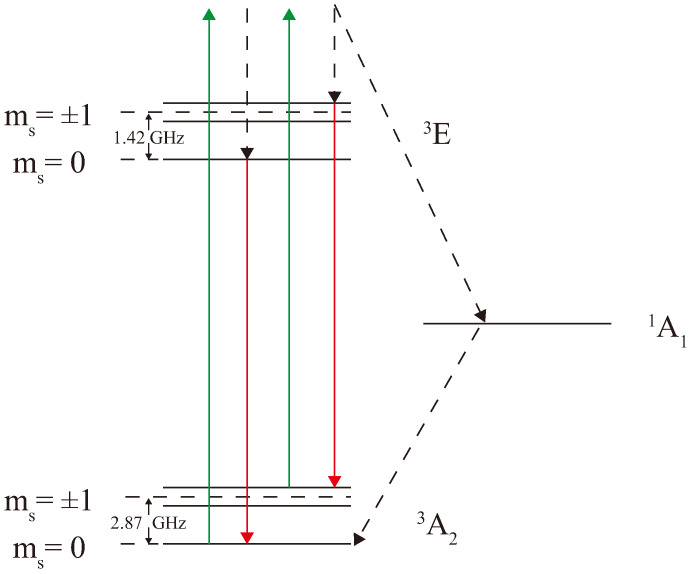
Schematic diagram of the NV center’s energy level structure. The green upward arrow extends into the phonon sideband above the excited state to represent off-resonant optical excitation (e.g., 532 nm), which is followed by rapid non-radiative relaxation to the lowest vibrational level of the excited state. The red downward arrow represents spontaneous fluorescence emission. The dashed lines indicate the non-radiative intersystem crossing (ISC) pathways via the metastable singlet states (A11 and E1) and internal non-radiative relaxations. The zero-field splitting (ZFS) for both the ground state (~2.87 GHz) and the excited state (~1.42 GHz) are indicated.

The nitrogen-vacancy (NV) center in diamond is a point defect exhibiting $C_{3v}$ symmetry. Both its ground state (A32) and excited state (E3) are spin-1 triplets (S=1), with the energy level structure illustrated in Figure 1 [20].

In the absence of an external magnetic field, the ground-state sublevels $|m_{s}=\pm 1\rangle$ are rendered degenerate and separated from the $|m_{s}=0\rangle$ level by zero-field splitting (ZFS) of D≈2.87 GHz at room temperature. Similarly, the excited-state sublevels $|m_{s}=\pm 1\rangle$ are rendered degenerate using a ZFS of approximately 1.42 GHz relative to the $|m_{s}=0\rangle$ level. Additionally, metastable singlet states (A11 and E1) exist between the ground and excited states.

Upon excitation from the ground state (A32) to the excited state (E3) using a 520 nm or 532 nm laser, relaxation occurs via two distinct pathways: (1) spin-conserving spontaneous emission, which creates fluorescence in the 637–800 nm range (Red); (2) non-radiative decay through the metastable singlet states via intersystem crossing (ISC), eventually returning to the ground state. Crucially, the $|m_{s}=0\rangle$ excited state decays primarily via the radiative pathway, whereas the $|m_{s}=\pm 1\rangle$ states exhibit a significantly higher probability of decaying via the non-radiative ISC pathway. Consequently, continuous laser illumination pumps the population from $|m_{s}=\pm 1\rangle$ into the $|m_{s}=0\rangle$ ground state, establishing optical spin polarization.

In parallel with the above, a resonant microwave field can manipulate the spin population between the $|m_{s}=0\rangle$ and $|m_{s}=\pm 1\rangle$ states. In the absence of a magnetic field, when the microwave frequency matches the ZFS (2.87 GHz), the population transfers from the $|m_{s}=0\rangle$ state to the “dark” $|m_{s}=\pm 1\rangle$ states, leading to a significant reduction in fluorescence intensity. In the presence of an axial magnetic field, the degeneracy of the $|m_{s}=\pm 1\rangle$ states is lifted due to the Zeeman effect, splitting them into $|m_{s}=+1\rangle$ and $|m_{s}=-1\rangle$ levels. By sweeping the microwave frequency while monitoring the fluorescence intensity, an Optically Detected Magnetic Resonance (ODMR) spectrum is obtained. This spectrum manifests as a single dip at 2.87 GHz in the zero field, or as two distinct dips centered around the ZFS frequency in the presence of a magnetic field.

Based on the physical processes described above, the ground-state Hamiltonian of the NV center can be expressed as

(1) $$H=D\left(S_{z}^{2}-S\left(S+1\right)/3\right)+\gamma_{e}\vec{B}\cdot \vec{S},$$

where D≈2.87 GHz represents the zero-field splitting parameter, and $\gamma_{e}\approx 28.03$ GHz/T is the electron gyromagnetic ratio. Additionally, S represents the dimensionless electron spin operator vector, and B is the applied external static magnetic field vector.

When an external bias magnetic field B is applied along the NV axis, the degenerate $m_{s}=\pm 1$ sublevels undergo Zeeman splitting. The resonance frequencies of the two transitions can be expressed as $\nu_{\pm }\approx D\pm \gamma_{e}B$. Traditional “side-peak locking” methods involve locking the microwave frequency to one of these explicitly split resonances (e.g., locking to $\nu_{-}$ or $\nu_{+}$). In this configuration, any fluctuation in the environmental magnetic field (ΔB) directly translates into a significant frequency drift of $\pm \gamma_{e}\Delta B$.

In contrast, our proposed “central-peak locking” approach utilizes an optimized weak magnetic field regime where the Zeeman splitting is sufficiently small. Under these conditions, the two split branches overlap and merge into a single, narrow central peak. For this merged feature, the effective central resonance frequency is $\nu_{0}\approx D$. Because $\nu_{0}$ is primarily governed by the zero-field splitting parameter, the central peak inherently exhibits a first-order immunity to external magnetic field variations. Furthermore, the overlapping of these near-degenerate transition states yields a composite resonance linewidth that is considerably narrower than that of individual widely split side peaks, thereby significantly enhancing the frequency discrimination slope.

Thus, for our system operating at the central peak, the intensity of the ODMR fluorescence signal I(ν) as a function of the microwave frequency ν can be approximately modeled as a single resonance feature [10,21]:

(2) $$I(\nu )=R\left[1-C\cdot L\left(\frac{\upsilon -\upsilon_{0}}{\Delta \upsilon }\right)\right],$$

Here, I(v) denotes the intensity of the ODMR fluorescence signal as a function of the applied microwave frequency v. R represents the baseline (off-resonance) photon count rate. $v_{0}$ is the central resonance frequency, and Δv represents the full width at half maximum (FWHM) of the spectral line. It should be noted that this parameter R remains consistent throughout the text, representing the same photon count rate used in calculations of shot-noise limits and total photon collection. C represents the contrast of the electron spin resonance (ESR), which corresponds to the normalized reduction in fluorescence intensity. Further, L(v) is the lineshape function of the resonance, typically approximated by a Lorentzian function:

(3) $$L\left(\frac{\upsilon -\upsilon_{0}}{\Delta \upsilon }\right)=1/[1+{\left(2(\upsilon -\upsilon_{0})/\Delta \upsilon \right)}^{2}],$$

**Figure 2:**
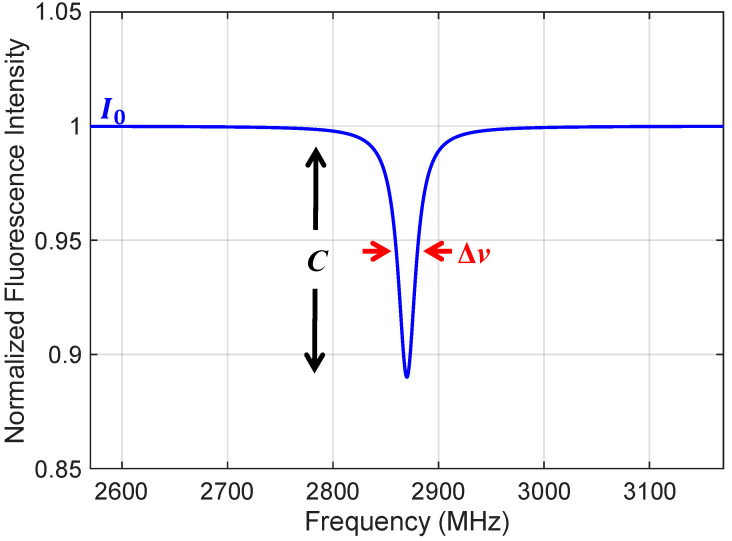
Simulation results of the ODMR Lorentzian lineshape (in zero magnetic field).The curve illustrates the fluorescence intensity as a function of the applied microwave frequency. Key spectral parameters governing the lineshape are highlighted, including the full width at half maximum Δv and the resonance contrast *C*.

Figure 2 presents the simulation results of the ODMR Lorentzian lineshape. It should be noted that the contrast C and the linewidth Δν in the figure will vary depending on the experimental configuration.

Assuming the measurement time is T, the total photon count is N=RT. Considering the system is dominated by photon shot noise with a standard deviation $\sigma_{I}=\sqrt{RT}$, $I_{0}$ denotes the off-resonance photon count rate, the signal-to-noise ratio (SNR) of the system can be defined as [14]

(4) $$SNR=\frac{S}{\sigma_{I}}=\frac{CI_{0}T}{\sqrt{RT}}.$$

Under continuous excitation by optical and microwave fields, the ground-state transition mechanism in NV centers is highly analogous to that utilized in dual-isotope Rubidium (Rb) frequency standards. By combining Equations (2) and (4), the frequency stability of the NV center in a CW-ODMR system, defined in terms of the Allan Deviation, can be derived and explicitly expanded as follows:

(5) $$\sigma \left(\tau \right)=\frac{1}{2\pi Q}\frac{1}{\left(SNR\right)}\frac{1}{\sqrt{\tau }}\propto \frac{\Delta v}{v_{0}\cdot C\sqrt{R\tau }}.$$

Here, Q represents the quality factor, defined as the ratio of the locking frequency v to the full width at half maximum (FWHM) Δν, and τ denotes the integration time. As explicitly shown in the expanded proportional relationship of Equation (5), the Allan deviation is directly proportional to the FWHM (Δv) and inversely proportional to both the contrast (C) and the square root of the photon count rate (R). However, since there is typically an inherent trade-off between contrast and linewidth (e.g., power broadening effects), optimization tailored to specific experimental conditions is critical.

### 2.2. Frequency Locking Principle Based on CW-ODMR

In the presence of continuous optical excitation and a microwave field with small-amplitude modulation, a combination of demodulation and PID control techniques is employed to generate an error signal that feeds back to the microwave frequency controller. By locking the microwave frequency to the ODMR peak, the system leverages the stable transitions defined by the fixed energy level spacing of the NV center to achieve a high-precision frequency standard.

To generate an error signal that is sensitive to frequency detuning, a small-amplitude sinusoidal modulation is applied to the microwave driving frequency:

(6) $$v\left(t\right)=v_{c}+v_{\mathrm{mod}}\cos\left(v_{m}t\right),$$

where $v_{c}$ denotes the microwave center frequency, $v_{mod}$ represents the modulation depth, and $v_{m}/2\pi$ is the modulation frequency. Under the condition that the modulation depth is significantly smaller than the ODMR linewidth, a first-order Taylor expansion can be performed on the fluorescence signal I around the center frequency $v_{c}$:

(7) $$I(t)\approx I(v_{c})+\frac{dI}{dv}|v_{c}\;v_{\mathrm{mod}}\cos(v_{m}t).$$

Evidently, the fluorescence signal contains a component at the same frequency as the modulation frequency $v_{m}$, with an amplitude proportional to the first derivative of the ODMR curve at $v_{c}$.

In the experiment, the detected fluorescence signal is multiplied by a reference signal (synchronized with the modulation) via a lock-in amplifier or a digital mixer, and the product is subjected to low-pass filtering to extract the DC component, yielding the demodulated error signal:

(8) $$e(t)=\mathrm{LPF}\left\{I(t)\cos(v_{m}t)\right\}\propto \frac{dI}{dv}\left|v_{c}\right..$$

Consequently, this error signal is proportional to the first derivative of the ODMR curve. When the microwave center frequency satisfies $v_{c}=v_{0}$, the error signal becomes zero, which corresponds precisely to the zero-crossing of the derivative at the resonance dip. This error signal is then fed into a PID (Proportional–Integral–Derivative) controller to adjust the microwave center frequency in real time. The control output can be expressed as

(9) $$\delta v_{c}(t)=K_{p}e(t)+K_{i}\int e\left(t\right)dt+K_{d}\frac{de(t)}{dt},$$

where $K_{p}$, $K_{i}$, and $K_{d}$ represent the proportional, integral, and derivative gain coefficients, respectively. Under closed-loop steady-state conditions, the system maintains the error signal e(t)≈0 via PID control, thereby keeping the microwave frequency continuously locked to the ODMR position, and enabling real-time tracking and control of the resonance frequency.

## 3. Experimental Setup and Method

### 3.1. Experimental Apparatus

**Figure 3:**
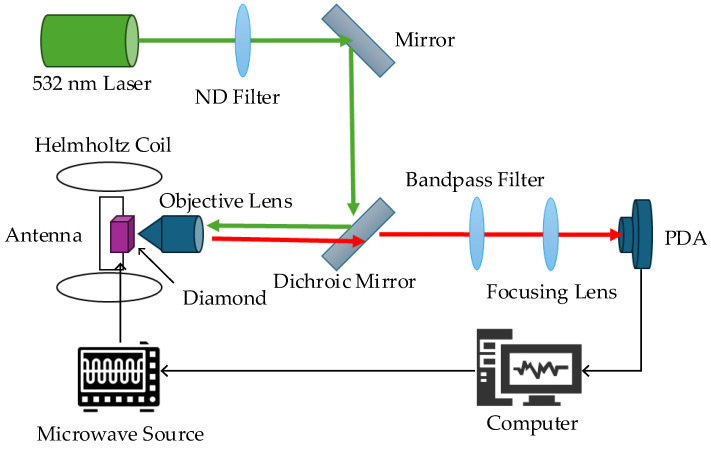
Schematic diagram of the experimental setup for the NV-center-based frequency locking system. The apparatus integrates a confocal microscopy optical path with microwave magnetic resonance control. Key components include a high-concentration diamond sample positioned within a custom three-axis Helmholtz coil, which regulates the static bias magnetic field to optimize the ODMR spectral characteristics. A coplanar waveguide antenna is utilized to efficiently couple the microwave driving field to the diamond surface for precise manipulation of the NV-center spin states. The green lines/arrows represent the excitation light, the red lines/arrows represent the fluorescence, and the black lines/arrows indicate signal transmission.

In this study, an experimental NV-center system based on a confocal microscopy optical path and microwave magnetic resonance control was constructed. The schematic of the experimental setup is illustrated in Figure 3.

The optical excitation section employed a 532 nm solid-state laser (CNI, Changchun, China, MLL-III-532) as the pump source, with a maximum output power of 300 mW. The laser beam first passed through an optical isolator (Maofeng Optoelectronics, Beijing, China, MFI0-3-532) to prevent back-reflections from interfering with the laser mode, thereby enhancing source stability. Subsequently, a neutral density filter (LBTEK, Changsha, China, NDFR-50C-6M-A) was used to adjust the excitation power, optimizing light intensity and avoiding thermal effects or signal saturation caused by excessive power. Reflected at a 45° angle by a dichroic mirror (Thorlabs, Newton, NJ, USA, DMLP567), the laser was directed into a high-numerical-aperture objective lens (Olympus, Tokyo, Japan, UPLSAPO 40 × 2) and focused inside the diamond sample. This objective lens system, combined with a pinhole structure at the front end of the detection path, formed a confocal microscopy system, significantly improving both the spatial resolution of excitation and the collection efficiency. In the detection path, the red fluorescence (wavelength range of approx. 637–800 nm) emitted by the NV centers was collected by the same objective lens and separated from the residual reflected pump light via the dichroic mirror. To further improve the signal-to-noise ratio (SNR), a bandpass filter (Thorlabs, Newton, NJ, USA, FBH650-40, center wavelength of 650 nm, bandwidth of 40 nm) was placed in the optical path to filter out stray background light. Finally, the processed fluorescence signal was detected by a high-sensitivity silicon avalanche photodetector (APD) (Thorlabs, Newton, NJ, USA, APD430A2/M). This detector features an effective active area of 13 mm^2^ and a bandwidth of DC-20 MHz, capable of converting the light intensity signal into a voltage signal in real time for transmission to a data acquisition (DAQ) card.

The diamond sample selected for the experiment was a 3.0 × 3.0 × 0.5 mm^3^ high-concentration NV-center ensemble sample (Element Six, Didcot, UK, DNV-B14) with a (100) primary polished face. It contained a substitutional nitrogen (N) concentration of approximately 13 ppm and an NV-center concentration of up to 4.5 ppm, with a natural ^13^C abundance of 1.1%. Macroscopically, the sample appeared deep purple, while microscopically, it exhibited good uniformity in color center distribution within the selected regions.

The microwave control system utilized a vector signal generator (Rhode & Schwarz, Munich, Germany, SMA100B) as the source. The generated microwave signal was amplified by a high-gain power amplifier (Mini-Circuits, New York, NY, USA, ZHL-16W-43-S+; max output of 16 W; gain of +42 dB at 2.87 GHz). To protect the microwave source from damage caused by reflected waves, a circulator (Qiyuan Microwave, Shenzhen, China, QY-CC-2/6-ST) was connected in series within the signal link to ensure unidirectional transmission. Ultimately, the microwave energy was efficiently coupled to the diamond sample surface via a coplanar waveguide antenna, forming a spatially uniform microwave driving field at the microscopic scale to achieve precise manipulation of the NV-center spin states. Additionally, to facilitate the regulation of ODMR spectral characteristics (such as the linewidth and contrast of the central peak), the system was equipped with a custom-made three-axis Helmholtz coil (CH-Magnetoelectricity Technology, Beijing, China, 3HLY16-10W). The coil assembly, with dimensions of 520 mm × 440 mm × 630 mm, provided a maximum static bias magnetic field of ±12 Gauss with uniformity of more than 0.5% in the central region, thus satisfying the requirements for precise weak-magnetic-field adjustments.

### 3.2. Locking Method

**Figure 4:**
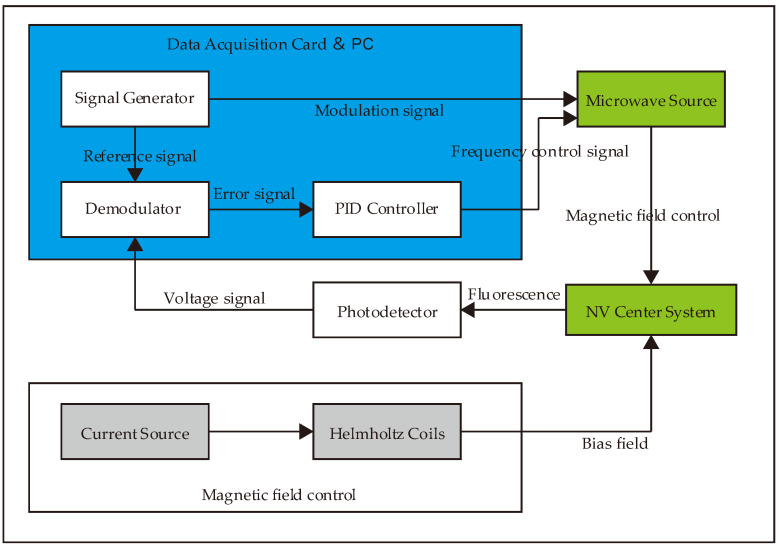
Block diagram of the microwave frequency locking scheme. The closed-loop control system utilizes continuous-wave optically detected magnetic resonance (CW-ODMR) to stabilize the 2.87 GHz microwave frequency. In this configuration, a small-amplitude sinusoidal modulation is applied to the microwave driving field. The resulting fluorescence signal is demodulated to generate an error signal, which is then processed by a proportional–integral–derivative (PID) controller. The PID output provides continuous feedback to the microwave frequency controller, locking the system specifically to the ODMR central peak.

To overcome the impact of environmental magnetic field fluctuations on the frequency standard, a closed-loop frequency locking technique based on the ODMR “central peak” was employed in this study. The control flow is illustrated in Figure 4.

The locking process primarily consists of two stages: error signal generation and feedback control. First, by adjusting the current of the Helmholtz coils, a specific bias magnetic field was applied to optimize the linewidth and contrast of the “central peak” in the ODMR spectrum, setting it to the optimal operating point. In our experiment, the optimal drive current was determined to be 50 mA, corresponding to a magnetic field of 50 μT. The system utilized a data acquisition card to generate a 10 kHz sinusoidal reference signal, which was split into two paths: one was used to apply a small-amplitude frequency modulation to the microwave source’s center frequency $v_{c}$ (experimentally set to a modulation depth of 500 kHz), while the other served as the reference signal for phase-sensitive detection.

In the closed-loop control phase, the fluorescence intensity—carrying the modulation signature—was detected by the APD and transmitted to a Dynamic Signal Acquisition and Generation device (NI-PXIe-7856R, National Instruments, Austin, TX, USA). This hardware platform functioned as the central processing unit, performing signal digitization and executing digital lock-in amplification to demodulate the input against the reference signal, thereby extracting an error signal proportional to the first derivative of the ODMR spectral profile. Subsequently, the error signal was processed by a digital PID (Proportional–Integral–Derivative) controller integrated within the PXIe-7856R module. The card computed the necessary feedback correction in real time and generated the control signal to adjust the microwave source’s output frequency, effectively compensating for any frequency drift. By configuring the polarity of the PID parameters, the system robustly locked the microwave frequency to the extremum of the ODMR central peak, ensuring a highly stable frequency output.

## 4. Results and Discussion

As indicated by Equation (1), the resonance frequency of the NV center is susceptible to interference from magnetic fields. Given the electron spin gyromagnetic ratio $\gamma_{e}/2\pi \approx 28.0$ GHz/T, a magnetic field fluctuation of 1 μT induces a frequency drift of 28 kHz. This magnetic sensitivity limits the frequency stability of the NV center.

**Figure 5:**
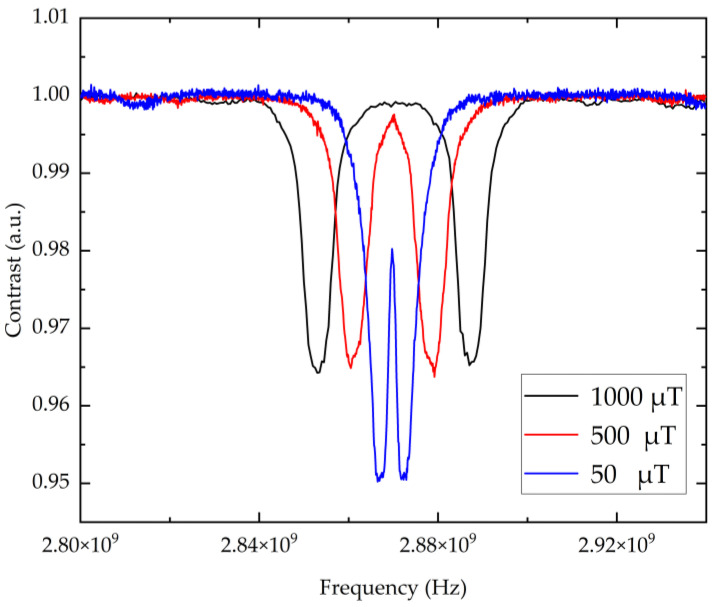
Optically detected magnetic resonance (ODMR) curves obtained under various bias magnetic fields. Under weak magnetic field conditions, the NV-center resonance peaks overlap to form a central peak. The experimental curves demonstrate the significant dependence of the central peak’s spectral characteristics on the applied magnetic field: as the bias field increases, the linewidth exhibits a distinct broadening effect, whereas the resonance contrast initially increases and subsequently saturates. These results illustrate the necessity of balancing contrast and linewidth to determine the optimal operating magnetic field (identified as 50 μT in this study) for the frequency locking system.

To address this issue, a frequency locking scheme based on the “central peak” of the ODMR spectrum is proposed, overcoming the limitations of the traditional method, which locks onto a single Zeeman-split resonance side peak. Under a weak magnetic field, the pair of NV-center resonance peaks overlap, forming a central peak whose center frequency is primarily determined by the ground-state zero-field splitting parameter D. The spectral characteristics (linewidth and contrast) of this central peak exhibit significant dependence on the magnetic field. As the applied bias magnetic field increases, the linewidth of the central peak exhibits a distinct broadening effect, while the contrast initially increases, followed by saturation, as shown in Figure 5. When the applied magnetic field is 50 μT, the central peak’s linewidth is approximately 1.8 MHz with a contrast of 3%. Upon increasing the field to 500 μT, the linewidth broadens to approximately 12 MHz due to increased Zeeman splitting, while the contrast rises slightly to 3.5%. With further increases in the magnetic field, the central peak broadens significantly due to Zeeman splitting; in addition, since the contrast stops increasing due to the physical limits (e.g., fluorescence collection efficiency) of the ODMR signal, the quality factor (Q-value) decreases.

Figure 5 indicates that the center frequency of the central peak is insensitive to magnetic field variations. This implies that when constructing an NV-center-based microwave frequency source, locking the frequency to the central peak can suppress the influence of environmental magnetic field noise on the output frequency without the need for complex magnetic shielding apparatus. Furthermore, in the low-magnetic-field region, the linewidth of the central peak (~1.8 MHz) is significantly superior to that of the side peak (~5 MHz). A narrower linewidth facilitates an enhanced frequency discrimination slope, thereby improving the long-term stability of the frequency source. However, according to Equation (4), the frequency locking stability depends on the signal-to-noise ratio (SNR), which is jointly influenced by linewidth and contrast; thus, a larger bias magnetic field is not necessarily better. The subsequent locking results under different magnetic fields also support this deduction. Therefore, contrast and linewidth must be accurately balanced to select the optimal operating point of the bias magnetic field. In this experiment, the optimal operating point was determined to be at an applied magnetic field of 50 μT.

It is important to explicitly discuss the practical limitations regarding this continuous reduction of the magnetic field. The primary advantage of the proposed method is that, under the optimized low-magnetic-field region (50 μT), the central peak possesses a significantly narrower linewidth (∼1.8 MHz) than the side peaks (∼5 MHz). This provides a steeper discrimination slope for stability enhancement. However, if the applied bias magnetic field becomes excessively weak (approaching zero), the Zeeman splitting decreases further, causing the split resonances to merge completely. While this maintains a narrow feature, it directly leads to a significant degradation of the central peak’s contrast. A central feature with such low contrast is difficult to detect robustly against background noise, which deteriorates the signal-to-noise ratio and consequently undermines the overall stability of the frequency standard. Therefore, the proposed central-peak locking method is valid specifically within an ‘optimized weak magnetic field’ regime (e.g., 50 μT). Operating at this carefully balanced point utilizes the narrow-linewidth advantage while avoiding the severe drop in central peak contrast associated with extremely weak fields, thereby ensuring the stable operation and robustness of the designed method.

To further experimentally verify the impact of magnetic field perturbations on different locking mechanisms, the frequency was locked to the central peak and the low-frequency side peak while applying a gradient magnetic field that changed in a stepwise manner over time. In the experiment, the driving current of the Helmholtz coils was adjusted to increase the applied bias magnetic field in increments of 20 μT every 100 s.

**Figure 6:**
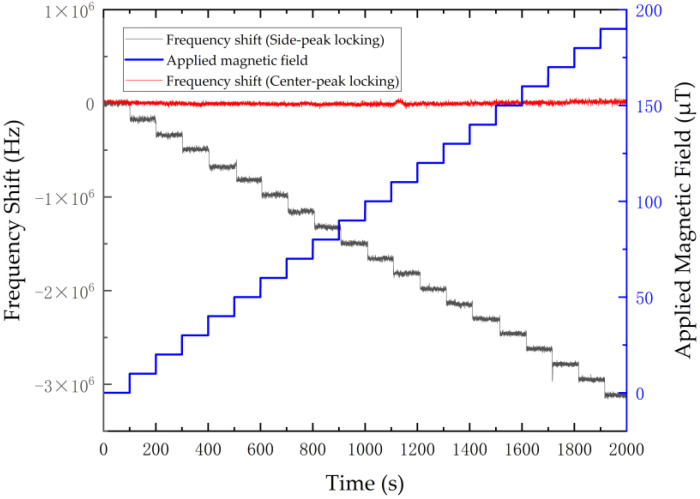
Comparison of microwave frequency variation under side-peak locking and central-peak locking schemes subjected to a continuously increasing stepwise magnetic field. The red line represents the relative frequency variation of the central-peak locking, which maintains only minute fluctuations around zero, demonstrating its excellent immunity to magnetic interference. In sharp contrast, the black line illustrates the locking frequency of the left side peak, which exhibits a significant linear drift toward lower frequencies due to the Zeeman effect as the magnetic field increases. This comparative experiment confirms that the central-peak locking strategy significantly suppresses frequency drift and is considerably more robust against external magnetic perturbations than conventional single side-peak locking.

The red line in Figure 6 represents the relative variation in the central-peak locking frequency over time. Despite the continuous stepwise increase in the applied magnetic field, the locking frequency of the central peak continues to exhibit only minute fluctuations around zero, demonstrating this locking mechanism’s excellent immunity to magnetic interference. In sharp contrast, as the magnetic field increases, the locking frequency of the left side peak (corresponding to the left resonance peak of the blue curve in Figure 5), represented as a black line, exhibits a significant linear drift in a low-frequency direction due to the Zeeman effect. This comparative experiment demonstrates that compared to single side-peak locking, the central-peak locking scheme is more robust in environments with a non-steady-state magnetic field; this is of significant engineering importance for achieving high-stability NV-center frequency sources.

**Figure 7:**
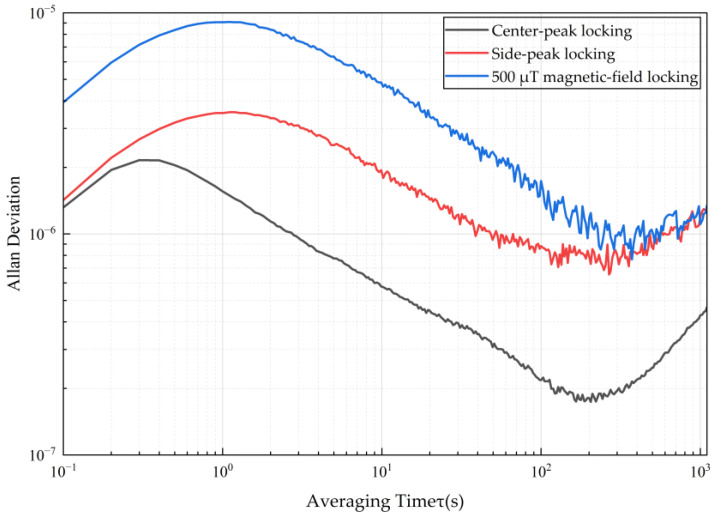
Allan deviation of the system output frequency as a function of integration time under different locking schemes and bias magnetic fields. The plot compares three distinct scenarios: locking to the central peak under an optimized weak magnetic field of 50 μT (black line), locking to a resonance side peak (red line), and locking to the central peak under a large bias magnetic field (blue line). The central-peak locking scheme under the optimized 50 μT field exhibits superior medium-to-long-term stability, reaching a minimum Allan deviation of approximately $1.73\times 10^{-7}$ at an integration time of 200 s. This represents a roughly fourfold improvement over the side-peak locking scheme. Furthermore, the blue curve illustrates that an excessive bias magnetic field degrades stability due to profound linewidth broadening induced by the Zeeman effect.

Figure 7 displays the test curves of the system output frequency’s Allan deviation as a function of integration time (τ) under different frequency locking positions and bias magnetic fields. The experiment compared three scenarios: locking to the central peak under an optimized weak magnetic field 50 μT (black line), locking to the resonance side peak (red line), and locking to the central peak under a large bias magnetic field (blue line). In the region of medium-to-long-term stability where integration time is τ=200 s, the central-peak locking scheme under the optimized magnetic field (black line) exhibits significantly better frequency stability than the side-peak locking scheme (red line). This result validates the deduction that under weak magnetic fields, the central peak possesses a higher quality factor (Q) due to its narrower resonance linewidth (approximately 1/3 of the side peak). Under identical SNR conditions, a narrower linewidth implies a steeper frequency discrimination slope, thereby enhancing the system’s frequency stability.

Specifically, at an integration time of τ=200 s, the Allan deviation of the central-peak locking reaches a minimum value of approximately $1.73\times 10^{-7}$, whereas that for the side-peak locking under equivalent conditions is $7.32\times 10^{-7}$. The central-peak locking scheme improves the medium-to-long-term stability of the system by a factor of approximately 4, proving the effectiveness of this scheme in enhancing the performance of NV-center frequency standards. It is worth noting that the results for the “large bias magnetic field” (blue line) indicate that a larger bias field is not always beneficial. When the applied magnetic field is excessive, the energy level splitting caused by the Zeeman effect causes significant broadening of the central peak’s linewidth (eventually exceeding the side peak’s linewidth), while the contrast ceases to increase due to the physical limitations of the system. This results in reduced sensitivity to frequency discrimination, causing the system’s frequency stability to become inferior to that under narrow-linewidth central-peak locking and, at certain timescales, side-peak locking. Furthermore, in the long-term, the Allan deviation curves for all locking schemes exhibit an upward trend to varying degrees after an integration time of τ>200 s. This is primarily attributed to long-term slow-drift mechanisms present in the NV system, including drift in the NV center’s zero-field splitting D caused by environmental temperature fluctuations and laser power instability. Theis long-term slow-drift noise limits the long-term stability of the frequency source, necessitating further research on optimizing temperature control and optical power stabilization systems.

**Figure 8:**
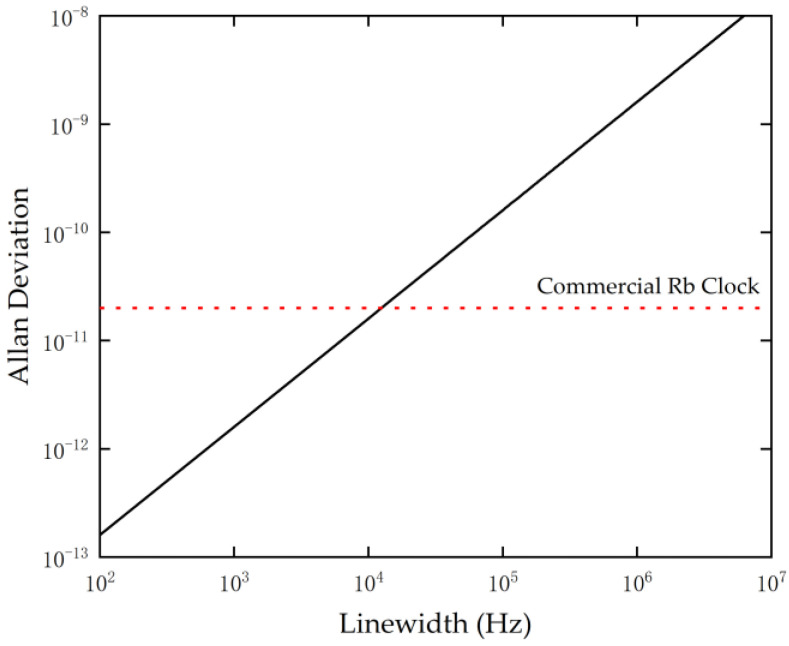
Relationship between theoretical Allan deviation (evaluated at τ=1 s) and ODMR linewidth. For comparison, the fractional frequency instability is 2 × 10^−11^ for a commercial Rb clock [25].

Upon incorporating the frequency stability formula (Equation (4)) derived earlier, it is evident that in the limit dominated by photon shot noise, the short-term stability $\sigma_{y}(\tau )$ of the NV-center frequency source is directly proportional to the magnetic resonance linewidth Δν and inversely proportional to the SNR (signal-to-noise ratio) (the product of contrast C and photon count rate R). This indicates that narrowing the linewidth of the ODMR spectral line is the most direct and effective pathway to enhancing the performance of the frequency source. Figure 8 displays the theoretical variation in frequency stability with ODMR linewidth. The ODMR linewidth of conventional diamond samples is typically limited to the MHz range due to inhomogeneous broadening of the electron spin resonance frequency caused by hyperfine interactions with the approximately 1.1% natural abundance of ^13^C isotopes (nuclear spin I=1/2). If the interference of the ^13^C spin bath is removed via isotopic purification growth techniques [22,23], combined with dynamical decoupling pulse techniques [24], the linewidth of the NV center can theoretically be narrowed to the kHz range or even lower. As shown in Figure 8, when the linewidth is narrowed to the kHz level, the theoretical stability of the NV-center frequency standard will improve by three orders of magnitude through this linear relationship, thereby reaching or even exceeding the performance levels of current commercial Rubidium atomic clocks.

For the system constructed in this experiment, photon shot noise limit estimation was performed by substituting the measured experimental parameters into Equation (4) and Equation (5). The following values were used: average photon count rate $R\approx 2.5\times 10^{11}$ cps (reference value, estimated based on actual light intensity), measured contrast C≈3%, and typical linewidth under current experimental conditions Δν≈1.8 MHz. By normalizing to an integration time of τ=1 s, the calculation yields a theoretical photon shot noise limit for this system of approximately $4.8\times 10^{-9}\tau^{-1/2}$, which represents the stability limit of the system in an ideal environment. Although the measured stability ($1.73\times 10^{-7}$ @ 200 s) does not reach this limit due to factors such as laser intensity noise, circuit noise, and thermal drift, the above analysis indicates that the system still holds tremendous potential for performance enhancement by improving linewidth characteristics [22,23].

While the central-peak locking method effectively suppresses magnetic field noise, other environmental factors, particularly potential thermal fluctuations, remain significant sources of frequency instability. For NV-center-based frequency standards, the zero-field splitting parameter (*D* ≈ 2.87 GHz) is temperature-dependent, with a coefficient of approximately dD/dT≈−74.2 kHz/K at room temperature. Since the central peak frequency is determined primarily by *D*, it remains susceptible to thermal drift. In our current experimental setup, passive thermal insulation was employed to mitigate rapid temperature variations. The observed long-term baseline drift in the Allan deviation (Figure 7) suggests that residual slow thermal fluctuations are likely the dominant instability source after the magnetic noise is suppressed. Future improvements will incorporate active temperature control (e.g., using Thermoelectric Cooling modules) and differential measurement techniques to further decouple thermal effects from the frequency standard output.

## 5. Conclusions

To address the issue of frequency drift caused by magnetic field fluctuations when using diamond NV centers as solid-state quantum frequency sources, this paper proposes a microwave frequency locking method based on the central peak of the ODMR spectrum. Through theoretical analysis of the NV-center energy level structure and experimental testing, the magnetic insensitivity of the central peak under weak magnetic fields was clarified. On this basis, a confocal optical path and a microwave control system were constructed. Using modulation and demodulation techniques, an error signal for feedback control of the microwave frequency was successfully extracted, achieving closed-loop locking of the 2.87 GHz microwave frequency to the NV-center transition energy levels.

This work indicates that, compared to the traditional side-peak locking scheme, frequency drift induced by magnetic field fluctuations is significantly suppressed with central-peak locking. The system’s short-term frequency stability (Allan deviation) was optimized to $1.73\times 10^{-7}$ at τ=200 s, verifying the robustness of this scheme in a non-magnetically shielded environment. This study not only experimentally confirms the feasibility of constructing high-stability microwave frequency sources based on NV centers but also lays a technical foundation for the future development of miniaturized, low-power, and interference-resistant chip-scale NV-center frequency standards. Future work will focus on further optimizing light collection efficiency and microwave circuit design, combined with temperature compensation techniques, to further enhance the medium-to-long-term stability of the NV-center frequency source.

## Data Availability

The authors declare the data that support the findings of this study are available upon reasonable request.
